# Influence of Popular Beverages on the Fracture Resistance of Implant-Supported Bis-Acrylic Resin Provisional Crowns: An In Vitro Study

**DOI:** 10.3390/polym15163411

**Published:** 2023-08-15

**Authors:** Oriana Karolina Ribera, José Manuel Mendes, Joana Mendes, Pedro Barreiros, Carlos Aroso, António Sérgio Silva

**Affiliations:** 1 Department of Oral Rehabilitation, Instituto Universitário de Ciências da Saúde, Rua Central da Gandra 1317, 4585-116 Gandra, Portugal; a26584@alunos.cespu.pt; 2 UNIPRO–Oral Pathology and Rehabilitation Research Unit, University Institute of Health Sciences (IUCS-CESPU), Rua Central da Gandra 1317, 4585-116 Gandra, Portugal; jose.mendes@iucs.cespu.pt (J.M.M.); joana.silva.mendes@iucs.cespu.pt (J.M.); pedro.barreiros@iucs.cespu.pt (P.B.);

**Keywords:** fracture resistance, CAD/CAM, polymethyl methacrylate, provisional restorations, dental implant

## Abstract

Implant-supported provisional restorations are critical for improving the esthetics and shaping of the peri-implant tissue. The mechanical properties of these provisional materials can be influenced by saliva, food, beverages, and interactions between these materials in the oral environment. Therefore, the integrity of provisional restorations should be preserved throughout the treatment period. This study aimed to evaluate the fracture strength of implant-supported polymethyl methacrylate (PMMA) provisional restorations made of computer-aided design and computer-aided manufacturing when immersed in different solutions at a controlled temperature of 37 °C for 7 days. Each analog-pillar-crown set was submerged in different liquids: 10 pieces were placed in distilled water then in tea, coffee, red wine, and Coca Cola^®^ for 1 week at a controlled oral temperature of 37 °C. The samples were then subjected to fracture forces. The moment of fracture of the crown was recorded and compared with those of the other samples. Specimens immersed in distilled water (control group) had the highest fracture resistance (mean [M] = 1331.00 ± 296.74 N), while those immersed in tea had the lowest mean resistance to fracture (mean [M] = 967.00 ± 281.86 N). Nutritional deficiency and inappropriate eating habits influence the fracture strength of temporary crowns, thereby rendering them more elastic or less resistant to fractures.

## 1. Introduction

Provisional implant-supported restorations are critical for improving esthetics, contouring, and shaping of the peri-implant tissue architecture. Dokania, R. et al. suggested the term “guided tissue healing” to describe the use of a provisional restoration for guiding soft-tissue architecture. This is a state-of-the-art approach in implantology [1,2], which also allows for rehabilitation during the period of osseointegration, as well as restores the function, phonetics, esthetics, and adaptation to the final shape of the restoration. The temporary restoration plays a fundamental role in oral rehabilitation [3,4].

The fracture resistance of temporary materials is generally low compared to permanent restorative material, such as ceramics; parafunctional habits, diet, and time are important factors that influence the fracture resistance. Strength should also be considered when selecting a temporary material for clinical use [3,5,6]. 

Strength tests are conducted to predict the fracture toughness of dental materials [7]. Since the strength of a material greatly influences its clinical performance [8,9], differences that are attributable to the effects of intraoral dietary solvents can be observed upon evaluating the strength of materials [5,10].

Fixed provisional restorations are fabricated using various resin-based materials [11]. Currently, there is no provisional material that meets the ideal requirements for all clinical situations. Dentists usually choose a product based on the ease of handling, esthetics, and cost [10,12]. Traditionally, polymethyl methacrylate (PMMA) has been available in a solvent/solute mixture form and has a chemical polymerization process [2]. The advantages of this type of material are its good marginal fit and transverse strength, thus providing a more durable restoration. PMMA has good polishability but low abrasion resistance [5,11].

Computer-aided design and computer-aided manufacturing (CAD/CAM) technology for the fabrication of temporary crowns allows for the shaping of materials with high precision, which cannot be easily achieved using a traditional method [13]. This process improves the physical properties of the materials over traditional methods [14,15]. CAD/CAM blocks are manufactured with 3D printing/additive manufacturing to create an interface that facilitates cementation on a titanium base, thus allowing the fabrication of screw-retained provisional restorations on the implant [16].

Some solvents, particularly those with acidic components, can penetrate the PMMA organic polymer network, thereby causing bulking and separation of the matrix filler phases, which is followed by softening of the polymers and chemical dissolution. In addition, the erosive effects of weak intra-oral acids (citric and lactic acids) on inorganic fillers can contribute to decreasing the hardness of materials such as Protemp or Luxatemp, which are composite materials containing Bis-GMA, TEGDMA, and glass- or silica-based fillers. Therefore, the chemical environment of the oral cavity may critically influence the degradation of the restorations [5,7].

The fracture resistance being generally low is a common cause for the failure of temporary restorations when compared to permanently supplemented materials; although they should be designed to avoid failure, they may still occur and cause discomfort to the patient. Thus, the mechanical strength of provisional crowns is important and should be considered to ensure clinical success while maintaining occlusal balance, preferably without the use of pontics [9,14].

This study aimed to evaluate the fracture strength of implant-supported PMMA provisional restorations made of CAD–CAM when immersed in different solutions (popular beverages) at a controlled temperature of 37 °C for 7 days. The specific objectives of this study were to evaluate the fracture resistance of temporary cemented crowns on implants to determine the influence of different solutions on the fracture resistance of temporary crowns, determine the solution that affects the fracture resistance of temporary crowns the most, and compare the qualitative results obtained in other studies. The null hypothesis was that the type of beverage does not influence the fracture resistance of the PMMA provisional implant-supported crowns, and that oral temperature does not influence the fracture of crowns immersed in different substances.

## 2. Materials and Methods

### 2.1. Materials 

All the materials used in this study were selected based on their importance and utility in dentistry, as well as their stability under normal use and storage conditions, which have been rated by clinical experience and longevity. The materials used to complete this study were the following: 60 identical PMMA crowns milled with a CAD–CAM 5-TECsystem (Zirkonzahn^®^, Gais, South Tyrol, Italy) 10 implant titanium abutments (Osteotech, Loures, Portugal) and 10 implant analogs (Osteotech, Loures, Portugal), Multilink automix system (Ivoclar^®^, Bremschlstraße, Bürs, Austria), and 5 aqueous solutions such as distilled water, green tea, coffee, red wine, and Coca Cola^®^ (Atlanta, GA, USA).

### 2.2. Methods

A laboratory protocol was established based on the International Organization for Standardization (ISO) 15850:2014 [17] and employed to test all the selected samples at the Oral Pathology and Rehabilitation Research Unit at the University Institute of Health Sciences (IUCS), CESPU, in Gandra, Portugal. 

#### 2.2.1. Preparation of the Sample

**Figure 1:**
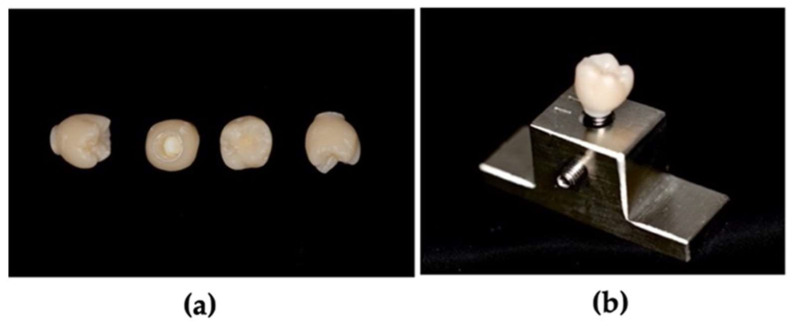
(**a**) PPMA crowns on CAD–CAM phases and (**b**) implant with PMMA crown replica, adapted to the support table. PMMA—polymethyl methacrylate; CAD–CAM—computer-aided design and computer-aided manufacturing.

Sixty identical PMMA crowns milled with a CAD–CAM 5-TEC system color A2–B2 (Zirkonzahn^®^, Gais, South Tyrol, Italy) were used in this study. They were designed by Zikonzahn software, cut with CAD/CAM Milling Bur PMMA Premium (6 mm), polished by diamond rubber wheels and pumice, and the high gloss was achieved with goat-hair brush and composite polishing paste. The tooth used for testing was 1.6. These provisional crowns (Figure 1a) were divided into five groups of 10 crowns each. Each crown was cemented on a titanium abutment with Multilink resin cement^®^ (Ivoclar^®^, Bremschlstraße, Bürs, Austria). All titanium abutments were screwed to an internal hexagon analog with a 4.1 platform (IPD^®^, Mataró, Barcelona, Spain). The analogs were adapted to a previously prepared titanium base (Figure 1b), which served as a support table to fixate in the testing machine: an Instron^®^ Electropuls E10000 Linear-Torsion (Norwood, MA, USA). The following aqueous solutions were used: distilled water, green tea, coffee, red wine, and Coca Cola^®^. The green tea, coffee, and red wine used were products from Portugal. The tea was prepared with 200 mL of hot water and 1 bag of green tea, the coffee was prepared with 200 mL of hot water and 3 spoons of instant coffee, and the amount of red wine was a glass.

The five groups with 10 provisional PMMA crowns (sample size calculations were performed with G*Power software (latest ver. 3.1.9.7; Heinrich-Heine-Universität Düsseldorf, Düsseldorf, Germany)) based on a minimum effect size of 0.50, α = 0.05, and 1-β = 0.80, screwed on the replicas, were submerged in the different aqueous solutions separately. Each group was exposed separately for 7 days in a thermostatic oven at 37 °C. Subsequently, all the crowns were subjected to force application at a constant rate until the occurrence of fracture. 

#### 2.2.2. Artificial Aging 

**Figure 2:**
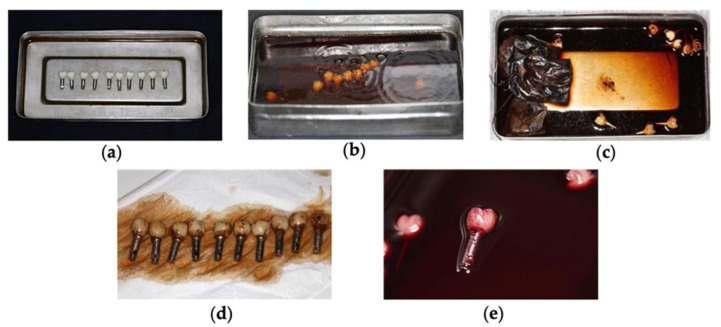
Artificial aging: (**a**) distilled water; (**b**) Coca Cola; (**c**) tea; (**d**) coffee; (**e**) red wine.

The five groups were separately subjected to artificial aging and immersed in different solutions for 7 days in a thermostatic oven at 37 °C, which is similar to the environment of the oral cavity. A temperature-controlled Memmert-Peltier-cooled incubator (Incubator IPP110 Plus, Schwabach, Germany) was used for artificial aging. Approximately 100 mL of different liquids were used on each sample (Figure 2) to ensure that all the crowns were completely submerged in the liquids and placed inside the oven. All drinks were the same during the storage time. 

### 2.3. Compression Test to Measure the Fracture Strength

The fracture strength of temporary materials is subject to the geometry of the restoration and the aging processes that occur in clinical applications. Although the laboratory values of flexural strength under static load may not reflect intra0oral behavior, these values are useful for comparing materials under controlled situations and can be a useful predictor of clinical performance [3,4].

**Figure 3:**
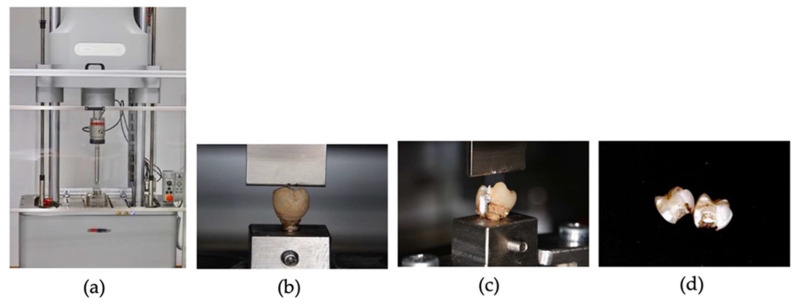
(**a**) Instron^®^ universal testing machine; (**b**) occlusal static load on a crown; (**c**) crown fracture (**d**) crowns after mechanical compressive strength test.

The Instron Electropuls E10000 LT (Norwood, MA, USA) EUA (Figure 3a) is a dynamic fatigue testing machine with a dynamic capacity of 10 KN, a static capacity of 7 KN, a stroke of 60 mm, a torque capacity of 100 Nm, a torsional stroke of 135°, and a diurnal aperture of 877 mm. A support table was attached to the machine for adaptation of the simulation structures to ensure that all the models were adjusted and produced equal compression. 

The test structures (analogs, interphases, and crowns) were screwed onto the titanium base using a torque wrench in the Instron universal testing machine (Electropuls E10000LT, Norwood, MA, USA) with a load cell having a capacity of 10,000 N. A traditional static load test per occlusal area of the crowns was applied in the central groove, which was progressively increased by 1 mm/min toward the structure until the occurrence of fracture (Figure 3b). 

The test results were transferred to WaveMatrix^®^ version 2.0 Dynamic Test software (Instron^®^, Norwood, MA, USA). This software allows users to define and run tests, as well as acquire data for a wide variety of dynamic and quasi-static applications. Thereafter, all the values and data were transferred to Microsoft Office Excel^®^, version 16.0 (Redmond, WA, USA), where statistical analysis of the obtained data was performed. 

### 2.4. Statistical Analysis 

Data were analyzed using a statistical computing program from Vienna, Austria (R Core team, version 4.2.2) [18]. Description of the variables was performed with the presentation of means (M) and standard deviations (SD), as well as box and line diagrams with lateral dispersion of the points using the jitter function. The comparison of the mean strength to fracture (N) and time to fracture (s) according to type of substance was performed using one-way analysis of variance. A comparison of the mean strength-to-fracture (N) according to substance type, while controlling for time-to-fracture (s), was performed using analysis of covariance. The sequential sum-of-squares method was used when the samples were balanced, that is, with the same number of elements. The effect size was calculated with η2 in the case of simple models and η2p in the case of multiple models, thus considering the following cutoff points: η2 < 0.06 (small effect), η2 < 0.14 (moderate effect), and η2 ≥ 0.14 (large effect), as well as η2p < 0.13 (small effect), η2p < 0.24 (moderate effect), and η2p ≥ 0.26 (large effect). The significance level considered was 5% [8,18].

## 3. Results

**Figure 4:**
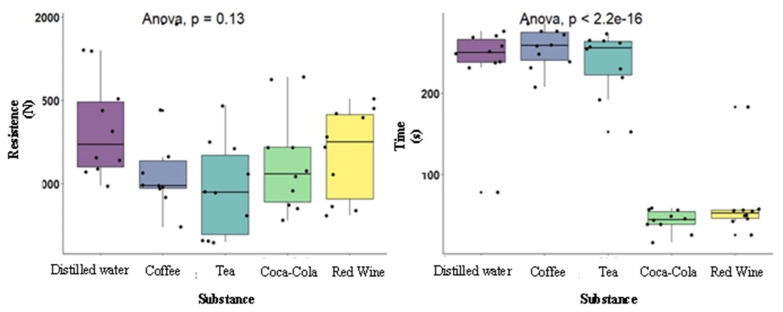
Box and line diagrams for the distribution of fracture toughness (N) and time-to-fracture (s) according to substance. ANOVA—analysis of variance.

**Figure 5:**
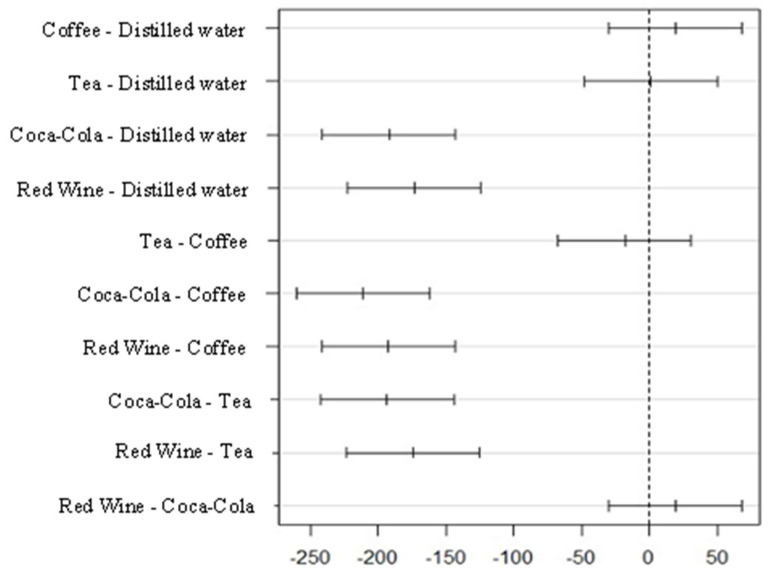
Tukey’s multiple comparison test for the time-to-fracture (s).

In this study, the fracture strength of the PMMA provisional crowns submerged in five substances, namely, distilled water, coffee, tea, Coca Cola^®^, and red wine, at a controlled oral temperature of 37 °C for 7 days were tested. Table 1 and Figure 4 present the results of the comparisons of the mean strength-to-fracture (N) and time-to-fracture (s) according to the substance type. Tea had the lowest mean strength-to-fracture (M = 967.00, SD = 281.86), and distilled water had the highest fracture resistance (M = 1331.00, SD = 296.74). However, no significant differences were observed when comparing the fracture strength according to the various types of substances [F(4,45) = 1.89 (*p* = 0.128), η2 = 0.14]. Moreover, the time-to-fracture was significantly associated with the substance analyzed [F(4,45) = 73.00 (*p* < 0.001), η2 = 0.87] with a high effect size. Tukey’s multiple comparison tests (Figure 5) revealed significant differences between the distilled water and Coca Cola (*p* < 0.001), the distilled water and red wine (*p* < 0.001), the Coca Cola and coffee (*p* < 0.001), the red wine and coffee (*p* < 0.001), the Coca Cola and tea (*p* < 0.001), and the red wine and tea (*p* < 0.001). The distilled water, coffee, and tea showed higher mean time until fracture compared with that of the Coca Cola and red wine. 

**Figure 6:**
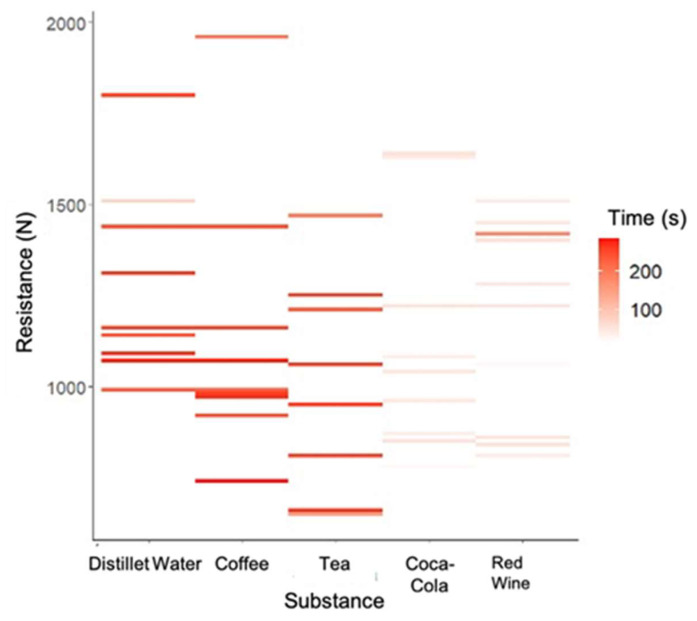
Heatmap for the association of strength-to-fracture (n), time-to-fracture (s) and substance.

Next, the interaction between the substance and time to fracture was evaluated based on the average force applied to the fracture. The results were significant [F(4,40) = 2.43 (*p* = 0.0636), η2p = 0.20], with a high effect size. Observation of the heatmap (Figure 6) led to the conclusion that the distilled water was conspicuous by consistently showing a higher strength and time-to-fracture in all the observations. Moreover, the tea presented a high mean time-to-fracture but lower fracture toughness. Coca Cola and red wine had the lowest mean time-to-fracture, while coffee had the highest mean-time to-fracture. 

## 4. Discussion

Provisional implant-borne rehabilitation aims to provide protection, stability, and function between definitive treatments [15]. During treatment plan development, provisional restorations are used to determine esthetic and functional effectiveness. The prognosis of a fixed restoration depends on the quality of the provisional restoration [19,20,21,22,23,24,25,26,27,28].

For successful treatment, a temporary material should meet the biological, mechanical, and esthetic requirements. The resistance to functional loads and retention forces are the mechanical factors that should be considered when selecting temporary restorative materials for clinical use. The fracture of temporary restorations is one of the most common factors, which is uncomfortable and costly to both patients and clinicians [19,21,22,23,24,25,26,27,28,29,30,31,32,33,34].

In the oral cavity, saliva, food, beverages, and the interactions between these liquids can degrade and age dental restorations, thereby causing changes in temporary restorations [21,22,24,29,31].

In this study, we tested the fracture resistance of implant-supported PMMA crowns submerged in different liquids (popular beverages) at a temperature of 37 °C for 7 days, with the aim of evaluating the fracture resistance of the crowns. 

According to Yanikoğlu et al., provisional restorations soften upon exposure to organic acids and various liquid food constituents. Furthermore, when provisional restorations are soaked in saliva, disintegration occurs at the interface; therefore, the chemical environment in the oral cavity can critically influence the in vivo degradation of restorations. The authors also reported that the specimens were stored for 14 days in different solutions (coffee without sugar, cola, and an energy-burning drink) and distilled water (control group) to partially simulate the oral environment [5]. In our study, we obtained similar results, which corroborated that those provisional restorations stored in coffee for 7 days demonstrated the lowest fracture resistance. Provisional crowns soften and become mostly elastic when the time-to-fracture decreases with exposure to various food and liquid constituents, such as tea and coffee (caffeine). 

The normal pH of the oral cavity is mainly neutral (pH = 7). Any food or drink that can reduce the pH in the mouth from 5.2 to 5.5 can cause penetration and softening of any type of restoration or prosthesis [21,22] For example, the destructive effect of alcohol is attributed to the softening of the polymeric matrix. Unlike distilled water, alcohol has greater permeability in composites owing to its chemical characteristics. Alcohol molecules can easily diffuse into the resin matrix, thus causing an increase in volume. In addition, fluid infiltration through the material occurs, which increases dissolution. Alcohol can reduce the longevity of a temporary restoration by two mechanisms: either by creating stress cracks and thus decreasing the fracture resistance, or by the corrosive effect of alcohol on the surface of the temporary crown, thereby accelerating fatigue fracture [22,24]. In our study, upon comparison with red wine, we found that alcohol not only directly influences the fracture resistance of temporary crowns, but also lowers the mean times to fracture, thus making them much more susceptible to fracture and less elastic. Sousa-Santos, S. et al. investigated the influence of oral pH on the fracture strength of implant-supported temporary restorations fabricated with two brands of bis-acrylic resins. In an attempt to simulate the behavior of restorative materials in the oral cavity and consider the influence of water absorption in bis-acrylic resins, this study used artificial saliva with pH values of 4 and 7 for 7 days in an incubator at 37 °C. The authors found no differences in fracture strength between the two brands of provisional crowns studied. The LuxaCrown^®^ (DMG^®^ Chemisch-Pharmazeutische, Hamburg, Germany) brand showed a higher fracture resistance than the Protemp™ 4 (3MTM St. Paul, MN, USA) brand. The saliva pH did not affect the fracture strength of the LuxaCrown^®^ provisional crowns, but it did affect the fracture strength of the Protemp™ 4 provisional crowns. The LuxaCrown^®^ provisional crowns, when exposed to artificial saliva with pH values of 4 and 7, exhibited a higher fracture strength than the Protemp™ 4 provisional crowns [3]. 

The surface hardness of a material is a complex mechanical property that is affected by several properties, including strength, proportional limit, ductility, malleability, and abrasion resistance. Diaz-Arnol et al. [19] demonstrated that the hardness number linearly correlates with the transverse strength and modulus of elasticity. These authors demonstrated no difference in the fracture strength of the self-curing provisional restorations after 48 h of storage in a moist or dry environment and stated that methacrylates are not cross-linked; without polymerization under pressure, air entrapment may occur, thus resulting in lower strength values [19]. This result does not coincide with that of our study but, it is in agreement with the results of the study by Poonacha et al., where the authors documented that, when exposed to aqueous environments, water absorption causes a subsequent mechanical deterioration of provisional restorations [23].

Although laboratory values of flexural strength under static load may not reflect intra-oral behavior, these values are useful for comparing materials under controlled situations and can be useful predictors of clinical performance [10,29]. 

Yanikoğlu, N.D. et al. evaluated test specimens that were conditioned in food-simulating liquids for 1 week prior to testing. This period could be considered a long duration, because temporary restorations come into contact with food and drinks only during eating and drinking until the teeth are cleaned [5]. However, these chemical agents can become trapped in the margins, pores of poorly handled materials, and connectors of poorly fabricated provisional restorations [9]. This process can provide a constant supply of chemical agents and a pathway for further diffusion into the restorative material, thereby resulting in faster degradation [19,35], which was confirmed in our study. Although laboratory values of the fracture strength under static load may not reflect intra-oral behavior, the liquids studied directly influenced the fracture strength of the provisional crowns.

Lang et al. [29] investigated the fracture resistance of temporary crown materials following 14 days of storage in distilled water and artificial aging. The authors observed low mechanical fracture behavior and total failure of the tested PMMA materials owing to deformation during the oral simulation. The authors also demonstrated that PMMA materials showed a water absorption up to 32 μg/mm, mainly owing to the polar properties of the resin molecules, which can act as plasticizers, thereby reducing the fracture resistance of the materials. We obtained similar results concerning the plasticity of crowns that were submerged for 7 days in tea or coffee, where the provisional crowns became more elastic with a longer time-to-fracture; however, the fracture resistance lowered because of caffeine [29].

Because temporary materials degrade under the influence of the environment in the oral cavity, it is important that patients be aware about the possible effects of alcohol and other substances on provisional restorations, especially if they need to be retained in the oral cavity for a long period [10,12,19].

Laboratory fatigue tests have been performed by subjecting a specimen to the application of alternating stresses below the yield point until fracture occurs. Despite their importance, they contain limitations that do not allow us to make a direct analogy with what happens in the oral cavity. In general, material loss is lower under clinical conditions than in laboratory studies. In addition, there has been a high variability of results, regardless of the type of study.

Trying to reproduce oral conditions in terms of temperature, humidity, occlusal loads, and microbial flora is still the biggest challenge for every researcher.

## 5. Conclusions

Based on the results obtained and the methodology described in this study, we formulated the following conclusions:

- The PMMA crowns immersed in different liquids exhibited mechanical properties that were capable of withstanding masticatory forces after aging. However, the laboratory values of the fracture strength under a static load may not reflect the intra-oral behavior, and it also must be considered that, in the oral cavity, the crowns are not immersed in 24 h solutions. The liquids used directly influence the fracture strength of temporary crowns.
- The pH, sugar, caffeine, and alcohol consumption are parameters that affect the structure, strength, and dissolution of temporary crowns, thereby making them less resistant to fracture and more elastic in the case of caffeine.
- Crowns submerged in distilled water (control group) had the highest mean fracture strength, and those in tea had the lowest mean strength-to-fracture, thereby making provisional crowns more elastic. Similarly, the tests with Coca Cola^®^ and coffee showed higher mean times-to-fracture of the provisional crowns.
- In our study, we achieved statistically significant results on the strength and time-to-fracture of the provisional crowns, where they became soft and elastic with exposure to tea, coffee, and Coca Cola^®^ (caffeine), which does not corroborate with the results of previous studies.

## Figures and Tables

**Table 1:** Univariate comparisons of fracture toughness (N) and time-to-fracture (s) according to the substance.

	Fracture Strength (N)		Time to Fracture (s)		
Substance	M	SD	M	SD	ANOVA
Distilled water	1331.00	296.74	235.66	57.24	Fracture Strength
Coffee	1122.00	345.18	254.81	24.09	F(4,45) = 1.89 (*p* = 0.128), η2 = 0.14
Tea	967.00	281.86	236.70	38.82	
Coca Cola	1129.00	304.46	43.352	13.86	Time to Fracture
Red wine	1185.00	272.53	62.432	43.52	F(4,45) = 73.00 (*p* < 0.001), η2 = 0.87

M—mean; SD—standard deviation; ANOVA—analysis of variance.

## Data Availability

The data that support the findings of this study are available from the corresponding author upon request.

## References

[1] Dokania R., Nayakar R., Patil R. (2015). Comparative evaluation of fracture resistance of three commercially available resins for provisional restorations: An in vitro study. Br. J. Appl. Sci. Tech..

[2] Mendes J.M., Botelo P.C., Mendes J., Barreiros P., Aroso C., Silva A.S. (2021). Comparison of fracture strengths of three provisional prosthodontic CAD/CAM materials: Laboratory fatigue tests. Appl. Sci..

[3] Sousa-Santos S., Silva A.S., Sousa-santos P., Vale T., Mendes T.M. (2023). The influence of saliva PH on the fracture resistance of two types of implant-supported bis-acrylic resin provisional crowns—An vitro study. J. Funct. Biomater..

[4] Alt V., Hannig M., Wöstmann B., Balkenhol M. (2011). Fracture strength of temporary fixed partial dentures: CAD/CAM versus directly fabricated restorations. Dent. Mater..

[5] Yanikoğlu N.D., Bayindir F., Apacay D.K., Beşir B. (2014). Flexural strength of temporary restorative materials stored in different solutions. Open J. Stomatol..

[6] Silva A.S., Martins D., de Sá J., Mendes J.M. (2021). Clinical evaluation of the implant survival rate in patients subjected to immediate implant loading protocols. Dent. Med. Prob..

[7] Delong R., Douglas W.H. (1983). Development of an artificial oral environment for the testing of dental restoratives: Bi-axial force and movement control. J. Dent. Res..

[8] Cohen J. (2013). Statistical Power Analysis for the Behavioral Sciences.

[9] Yesilyurt C., Yoldas O., Altintas S.H., Kusgoz A. (2009). Effects of food-simulating liquids on the mechanical properties of a silorane-based dental composite. Dent. Mater. J..

[10] Yap A.U.J., Mah M.K.S., Lye C.P.W., Loh P.L. (2004). Influence of dietary simulating solvents on the hardness of provisional restorative materials. Dent. Mater..

[11] Silva A.S., Carvalho A., Barreiros P., de Sa J., Aroso C., Mendes J.M. (2021). Comparison of fracture resistance in thermal and self-curing acrylic resins—An in vitro study. Polymers.

[12] Preis V., Hahnel S., Behr M., Rosentritt M. (2018). In vitro performance and fracture resistance of novel CAD/CAM ceramic molar crowns loaded on implants and human teeth. J. Adv. Prosthodont..

[13] Abdullah A.O., Tsitrou E.A., Pollington S. (2016). Comparative in vitro evaluation of CAD/CAM vs conventional provisional crowns. J. Appl. Oral Sci..

[14] Karaokutan I., Sayin G., Kara O. (2015). In vitro study of fracture strength of provisional crown materials. J. Adv. Prosthodont..

[15] Rayyan M.M., Moushelib M., Sayed N.M., Ibrahim A., Jimbo R. (2015). Comparison of interim restorations fabricated by CAD/CAM with those fabricated manually. J. Prosthet. Dent..

[16] Magne P., Carvalho A.O., Bruzi G., Giannini M. (2015). Fatigue resistance of ultrathin CAD/CAM complete crowns with a simplified cementation process. J. Prosthet. Dent..

[17] (2014). Plastics — Determination of tension-tension fatigue crack propagation — Linear elastic fracture mechanics (LEFM) approach.

[18] R Core Team (2022). R: A Language and Environment for Statistical Computing.

[19] Diaz-Arnol A.M. (2008). Flexural and fatigue strengths of denture base resin. J. Prosthet. Dent..

[20] Guler A.U., Yilmaz F., Kulunk T., Guler E., Kurt S. (2005). Effects of different drinks on stainability of resin composite provisional restorative materials. J. Prosthet. Dent..

[21] Cândido M. (2017). Estudo da Erosão Dentária Provocada Pelo Consumo de Coca-Cola Utilizando Espetroscopia Raman e de Fluorescência de Raios-X. Ph.D. Thesis.

[22] Fatemi F.S., Vojdani M., Khaledi A.A.R. (2019). The effect of food-simulating agents on the bond strength of hard chairside reline materials to denture base resin. J. Prosthodont..

[23] Poonacha V., Poonacha S., Salagundi B., Rupesh P.L., Raghavan R. (2013). In vitro comparison of flexural strength and elastic modulus of three provisional crown materials used in fixed prosthodontics. J. Clin. Exp. Dent..

[24] Gultekin P., Gultekin B.A., Aydin M., Yalcin S. (2013). Cement selection for implant-supported crowns fabricated with different luting space settings. J. Prosthodont..

[25] Farzin M., Torabi K., Ahangari A.H., Derafshi R. (2014). Effect of abutment modification and cement type on retention of cement-retained implant supported crowns. J. Dent..

[26] Proussaefs P. (2015). Immediate provisionalization with a CAD/CAM interim abutment and crown: A guided soft tissue healing technique. J. Prosthet. Dent..

[27] Rosentritt M., Hahnel S., Engelhardt F., Behr M., Preis V. (2017). In vitro performance and fracture resistance of CAD/CAM-fabricated implant supported molar crowns. Clin. Oral Investig..

[28] Gujjari A.K., Bhatnagar V.M., Basavaraju R.M. (2013). Color stability and flexural strength of poly (methyl methacrylate) and bis-acrylic composite based provisional crown and bridge auto-polymerizing resins exposed to beverages and food dye: An in vitro study. Indian J. Dent. Res..

[29] Lang R., Rosentritt M., Behr M., Handel G. (2003). Fracture resistance of PMMA and resin matrix composite-based interim FPD materials. Int. J. Prosthodont..

[30] Nicodemo C., Rezende C., Moretti-Neto R., Rubo J. (2013). Micro-hardness of acrylic resin utilized for provisional crowns: Effect of different polymerization techniques and pHcycling. Braz. Dent. Sci..

[31] Pytko-Polonczyk J.J., Jakubik A., Przeklasa-Bierowiec A., Muszynska B. (2017). Artificial saliva and its use in biological experiments. J. Physiol. Pharmacol..

[32] Muley B.Y., Shaikh S.R., Tagore M.M., Khalikar A.N. (2014). Effect of dietary simulating solvents on the mechanical properties of provisional restorative materials—An in vitro study. J. Indian. Prosthodont. Soc..

[33] Levartovsky S., Peleg G., Matalon S., Tsesis I., Rosen E. (2022). Maximal bite force measured via digital bite force transducer in subjects with or without dental implants—A pilot study. Appl. Sci..

[34] Singh A., Garg S. (2016). Comparative evaluation of flexural strength of provisional crown and bridge materials—An in vitro study. J. Clin. Diagn. Res..

[35] Rajaee N., Vojdani M., Adibi S. (2014). Effect of food simulating agents on the flexural strength and surface hardness of denture base acrylic resins. Oral Health Dent. Manag..

